# Xylan-Based Hydrogels as a Potential Carrier for Drug Delivery: Effect of Pore-Forming Agents

**DOI:** 10.3390/pharmaceutics10040261

**Published:** 2018-12-05

**Authors:** Minmin Chang, Xinxin Liu, Ling Meng, Xiaohui Wang, Junli Ren

**Affiliations:** State Key Laboratory of Pulp and Paper Engineering, South China University of Technology, 510640 Guangzhou, China; 17728109456@163.com (M.C.); lxx19910312@163.com (X.L.); mengling0117@163.com (L.M.); fewangxh@scut.edu.cn (X.W.)

**Keywords:** carboxymethyl xylan, hydrogel, pore-forming agents, drug delivery

## Abstract

Pore-forming agents have a significant influence on the pore structure of hydrogels. In this study, a porogenic technique was employed to investigate the preparation of macroporous hydrogels which were synthesized by radical copolymerization of carboxymethyl xylan with acrylamide and *N*-isopropylacrylamide under the function of a cross-linking agent. Six kinds of pore-forming agents were used: polyvinylpyrrolidone K30, polyethylene glycol 2000, carbamide, NaCl, CaCO_3_, and NaHCO_3_. The application of these hydrogels is also discussed. The results show that pore-forming agents had an important impact on the pore structure of the hydrogels and consequently affected properties of the hydrogels such as swelling ratio and mechanical strength, while little effect was noted on the thermal property of the hydrogels. 5-Fluorouracil was used as a model drug to study the drug release of the as-prepared hydrogels, and it was found that the drug release was substantially improved after using the NaHCO_3_ pore-forming agent: a cumulative release rate of up to 71.05% was achieved.

## 1. Introduction

Hydrogels are three-dimensional networks composed of cross-linked polymers which are insoluble in water and can absorb a large amount of water [1]. Recently, hydrogels have attracted extensive interest in some biomedical fields such as drug delivery, tissue engineering, and artificial muscles [2,3,4]. However, most hydrogels have a low swelling rate and take a long time to reach their equilibrium swelling state, which limits the application of hydrogels. Hydrogels with porous structure have attracted lots of attention due to their large pore size and surface area [5]. A porogenic technique could produce desirable pore sizes within the hydrogel network. Pore-forming agents are added during the hydrogel preparation and removed after polymerization to form the pore structure and control the pore size of hydrogels [6,7]. For example, a kind of macroporous polyacrylamide (PAM) hydrogel for protein immobilization was developed using poly(ethylene glycol) (PEG) as a pore-forming agent. The results showed that PEG could enhance macromolecule diffusion into the interior of the hydrogel and that this hydrogel could be used in tissue engineering and protein arrays [8]. Stable alginate hydrogels were fabricated with controlled pore dimensions in the range of 5–50 µm by sacrificial CaCO_3_ templates [9]. A kind of DNA–polymer hybrid hydrogel was prepared using PEG as the pore-forming agent [10]. It was found that the pore size of the hydrogel increased with increasing PEG content. The pore size distribution of the altered hydrogels could enhance the permeability of the materials, resulting in faster hydrogel equilibration. Accordingly, pore-forming agents could be used to control the pore size of hydrogels [11].

With the deterioration of the environment and the shortage of resources, natural polymers such as cellulose, chitosan, collagen, and hemicellulose have been used for the preparation of hydrogels [12,13,14]. The introduction of natural polymers endows hydrogels with biocompatibility and biodegradation [15]. Xylan is a kind of hemicellulose with a backbone of β-(1→4) linked d-xylosyl residues substituted with l-arabinofuranosyl, 4-*O*-methyl-d-glucuronopyranosyl, l-galactopyranosyl, or d-glucuronopyranosyl units at C-2 and/or C-3 of the main chain [16]. Xylan also possesses some medical properties such as immunological defense, anticancer, and antioxidant properties [17]. Due to the stability of xylan in human stomach and intestine, it has great potential as a drug release carrier [18]. A few studies have focused on xylan-based hydrogels. A xylan-based hydrogel was prepared by the cross-linking polymerization of xylan with *N*-isopropylacrylamide (NIPAm) and acrylic acid (AA) under UV irradiation [19]. This hydrogel was further used for acetylsalicylic acid release. The cumulative release rate of acetylsalicylic acid could reach up to 90.5% after sustained release for 5 h in simulated intestinal fluid. A new photoresponsive hydrogel was prepared by free radical copolymerization of xylan-type hemicellulose methacrylate with 4-[(4-acryloyloxyphenyl)azo]benzoic acid (AOPAB) [20]. These hydrogels showed responsive behavior to light, and vitamin-B12-loaded hydrogel showed a high drug cumulative release rate under UV irradiation. A simultaneous in situ formation of xylan nanohydrogels and encapsulation of horseradish peroxidase (HRP) was achieved through the selective hydrolysis of oat spelt xylan by recombinant α-l-arabinofuranosidase (AbfB) with polymeric xylan substrate specificity [21]. These hydrogels could release the encapsulated HRP in its active form over a period of 180 min, indicating that these hydrogels could potentially be used as an encapsulation matrix for the targeted delivery and slow release of bioactive substances. A new pH-sensitive and biodegradable hemicellulose-based hydrogel was prepared by grafting AA into hemicellulose [22]. The cumulative release rate of the hydrogels loaded with acetylsalicylic acid could reach up to 85% after 6 h. Therefore, xylan is promising for applications in drug delivery.

5-Fluorouracil (5-Fu) is a kind of hydrophilic anticancer drug used in clinics and is employed in systemic chemotherapy of solid tumors such as breast, colorectal, liver, and brain cancer [23]. 5-Fu inhibits the synthesis of DNA to a lesser extent over a longer period [24]. Continuous administration of 5-Fu maintains its therapeutic activity via metabolism. However, excessive concentrations of 5-Fu would cause a toxic effect. Thus, it is necessary to control a low concentration and sustained release [25].

The pore structure of hydrogels has an important influence on their properties such as their swelling ratio and mechanical properties which would affect their application. Porogenic techniques are an effective method to produce different pore structures in hydrogels and are suitable for different applications such as a culture medium for marine bacteria or drug release. Some reagents have been used as pore-forming agents, including organic reagents and inorganic reagents such as PEG and CaCO_3_ mentioned above. Very important for the selection of pore-forming agents is that the agents should have a good pore-forming effect and have no negative impact on the material properties. However, few studies have focused on the impact of different pore-forming agents on hydrogels, especially for xylan-based hydrogels. In this work, six different organic and inorganic pore-making agents with various molecular weights were applied to study their influence on the properties of xylan-based hydrogels such as the pore structure, swelling ratio, mechanical properties, and thermal stability. The preparation of xylan-based hydrogels was conducted by radical copolymerization of carboxymethyl xylan (CMX) with acrylamide (AM) and NIPAm under the function of cross-linking agents. During the preparation of hydrogels, six kinds of pore-forming agents were used: polyvinylpyrrolidone K30 (PVP), polyethylene glycol 2000 (PEG2000), carbamide, NaCl, CaCO_3_, and NaHCO_3_. These hydrogels were characterized by Fourier transform infrared (FTIR), scanning electron microscopy (SEM), and thermogravimetric analysis (TGA). The application of the as-prepared hydrogels as drug carriers for 5-Fu delivery was also investigated.

## 2. Materials and Methods

### 2.1. Reagent and Apparatus

Sodium chloroacetate (>99.6%) was purchased from Aladdin (Shanghai, China). Bagasse xylan (M_w_ of 49,000 g·mol^−1^, xylose > 85%) was purchased from Yuanye (Shanghai, China). NaOH, carbamide, absolute ethyl alcohol, and glacial acetic acid were purchased from Jinhuada Chemical Reagent Factory (Guangzhou, China). AM, ammonium persulfate (APS), *N*,*N*’-Methylenebis(2-propenamide) (MBA), and ZnCl_2_ were purchased from Fuchen Chemical Reagent Factory (Tianjin, China). NIPAm, *N*,*N*,*N*,*N*-tetramethylethylenediamine (TEMED), PEG2000, phosphate buffer (PBS, pH = 7.4), and 5-Fu were purchased from MACKLIN (Shanghai, China). Hydrochloric acid, NaCl, CaCO_3_, and NaHCO_3_ were purchased from Guangzhou Chemical Reagent Factory (Guangzhou, China). Polyvinyl pyrrolidone (PVP, M_w_ of 40,000) was purchased from Richjiont (Shanghai, China). Ultra-water was used in the preparation of hydrogels.

The thermostatic oscillator was used in drug release (TH7-C, Taicang Experimental Equipment Factory, Jiangsu, China). Microwave catalytic synthesizer was used to synthesize carboxymethyl xylan (XH-300UL, Beijing Xianghu Science and Technology Development Co. LTD, Beijing, China). Analytical balance was purchased from Sartorius Scientific Instruments (Beijing) Co. LTD (BSA224S-CW, Beijing, China). Heating agitator was used to control the reaction temperature (RCT basic, IKA, Staufen, Germany). UV spectrophotometer was used to detect the absorbance (UV-1800, Shimadzu, Kyoto, Japan). Vacuum drying chamber was purchased from Shanghai Yiheng Scientific Instrument Co. LTD (BPZ, Shanghai, China). Freeze-drying machine was purchased from Virtis (FM25XL-70, SP Scientific, Warminster, PA, USA).

### 2.2. Synthesis of Carboxymethyl Xylan (CMX)

CMX was synthesized under microwave irradiation according to our previous work [26]. Briefly, 6.6 g of xylan was added to 25 mL of ultra-pure water and was heated and stirred in an 80 °C oil bath. After 70 min, the xylan was completely dissolved. After the xylan solution cooled down to room temperature, 1.0 g of sodium hydroxide dissolved in 2 mL ultra-pure water was added. After 20 min, 11.648 g sodium chloroacetate (dissolved in 11 mL ultra-pure water) and 1.0 g sodium hydroxide (dissolved in 2 mL ultra-pure water) were added to the reaction system for 20 min. Then, the reaction was transferred to a microwave reactor at a power of 300 W and a temperature of 65 °C for 20 min. After the reaction was over, the system was cooled down and neutralized using acetic acid. The dialysis was conducted for one week at room temperature. At last, the sample was freeze-dried to get the sample powder and put into the dryer. The obtained CMX had a substituting degree of 0.14. The calculation method was obtained from the literature [27].

### 2.3. Peparation of Hydrogels

Xylan-based hydrogel was prepared by the following steps: Quantities of 0.5 g of prepared CMX, 4.5 g of AM, and 0.1 g of NIPAm were dissolved in 40 mL of ultra-pure water with a magnetic stirrer at 1 °C for 15 min. N_2_ was bubbled into the system to remove oxygen during this process. Quantities of 0.05 g each of MBA and APS were added, and then 0.25 g of the pore-forming agent was added as well as 50 μL TEMDA. Subsequently, the homogeneous solution was poured into two molds (with a diameter of 30 mm and a height of 15 mm). After forming the gels, the mold was transferred to the refrigerator for 24 h for further reaction. Then, the hydrogels were immersed in ZnCl_2_ solution (0.05 mol/L) for one day to enhance the hydrogels’ strength by metal coordination bonding, and different methods were applied to remove the pore-forming agents and unreacted chemicals; these are shown in Table 1. Some of the samples were cut into slices (about 8 mm length, 5 mm width, and 3mm thickness) and dried in a freeze-dryer. The dried samples were stored in a dryer, and the wet untreated samples were put in the refrigerator before testing.

### 2.4. Characterization of Hydrogels

Hydrogels were characterized by their FTIR spectra (TENSOR27, Bruker, Germany). These spectra were collected from KBr-based discs. Each spectrum was obtained in the range of 4000–400 cm^−1^ at a resolution of 4 cm^−1^ and recorded for a total of 32 scans. Before testing, hydrogels were ground into powder with a mortar. Then, the 1% finely ground hydrogels were mixed with KBr to be pressed into a plate for measurement.

The mechanical properties of the hydrogels were measured at room temperature using a universal testing machine (INSTRON 5565, Instron Corpration, Boston, MA, USA). As-prepared hydrogels with a cylindrical shape were placed at the center of the lower compression plate. The upper plate then compressed the samples at a velocity of 3 mm·min^−1^ until the hydrogels were broken.

A thermal gravimetric analyzer (TA Q500, TA Instruments, Newcastle, DE, USA) was used to evaluate the thermostability of the hydrogels prepared by different pore-forming agents. Samples of 8–10 mg of the hydrogels were transferred to a Pd plate. The temperature was increased from room temperature to 700 °C at a 10 °C/min heating rate in an open Pd plate. The thermal analyses were conducted under a dry nitrogen atmosphere with a flow rate of 25.0 mL/min.

SEM (EVO 18, Carl Zeiss Jena, Germany) was used to observe the surface morphology of hydrogels at an acceleration voltage of 10 KV. Dried hydrogels were cut into thin sheets with a blade. Before testing, hydrogel samples were fixed on a metal stub and coated with gold to increase the conductivity of the samples.

Electronic probe energy-dispersive spectrometry (EDS) (EPMA-1600, Shimadzu, Kyoto, Japan) was used to confirm the presence of Zn^2+^ in hydrogels. The dry sheet sample was sprayed with gold before testing.

An atomic absorption spectrometer (Z-2000, Hitachi, Tokyo, Japan) was used to detect the content of zinc in the hydrogels. A certain weight of dry hydrogel was transferred to 0.5 mol/L HCl solution (10 mL), and the mixture was stirred at room temperature for 8 h. The zinc content of the eluent liquid was detected by atomic absorption spectrometry. The zinc content was expressed as percentage of mass, which was calculated using the following formula:

W = C × V/M × 100%(1)

where C is the mass concentration of zinc, V is the volume of solution, and M is the weight of the dry hydrogel.

### 2.5. Swelling Behavior Studies of Hydrogels

The swelling behavior of hydrogels was evaluated by the gravimetric method. The weight of the dry hydrogel was recorded firstly, and then the dry hydrogel was immersed in PBS solution at room temperature. After a fixed time, the swollen hydrogel was removed from water, dried with clean filter paper, and weighed. The swelling ratio (SR) and the equilibrium swelling ratio (Seq) were calculated using the following formulae [22]:

SR = (W_t_ − W_d_)/W_d_ (2)

S_eq_ = (W_eq_ − W_d_)/W_d_ (3)

where W_t_ is the weight of the swollen hydrogel, W_d_ is the initial weight of the dried hydrogel, and W_eq_ is the equilibrium weight of the swollen hydrogel.

### 2.6. Drug Release

#### 2.6.1. Standard Curve of 5-Fu

For the method of drug release, we refer the reader to the published literature [28]. The standard curve of 5-Fu was obtained firstly. 5-Fu solution with a concentration of 0.233 mg/mL was accurately prepared. Then, 0.50, 1.00, 1.50, 2.00, 2.50, 3.00, or 3.50 mL of the solution was added to a 50.00 mL volumetric flask. The absorbance of these solutions was measured at 265 nm using an ultraviolet spectrophotometer. Finally, the standard working curve of 5-Fu was obtained (A = 0.0527C − 0.0006, R^2^ = 0.9995).

#### 2.6.2. Preparation of 5-Fu-Loaded Hydrogels

The aqueous solution of 5-Fu (0.8072 mg/mL) was prepared first. Dried hydrogels were placed in this 5-Fu solution at 20 °C. After 2 days, the hydrogels were removed from PBS, and the hydrogel surface was flushed with a small amount of deionized water to remove the unloaded drug. The volume of the mixture of residual liquid and flushing fluid was accurately measured. Then, the absorbance of the mixture was measured. According to the absorbance of the solution and standard curve, the weight of 5-Fu in the hydrogels was calculated. Finally, 5-Fu-loaded hydrogels were dried to a constant weight in a vacuum.

#### 2.6.3. Drug Release of Hydrogels

Hydrogels loaded with 5-Fu were added to a PBS buffer solution (pH = 7.4) at 37 °C. Then, they were placed into a 37 °C thermostatic oscillator (100 r/min) and were sampled at regular intervals. The absorbance of the solution was determined by ultraviolet spectrophotometry at 265 nm. According to the standard working curve, the concentration of 5-Fu was calculated.

## 3. Results and Discussion

### 3.1. Pore-Making Mechanism for Hydrogels

**Scheme 1:**
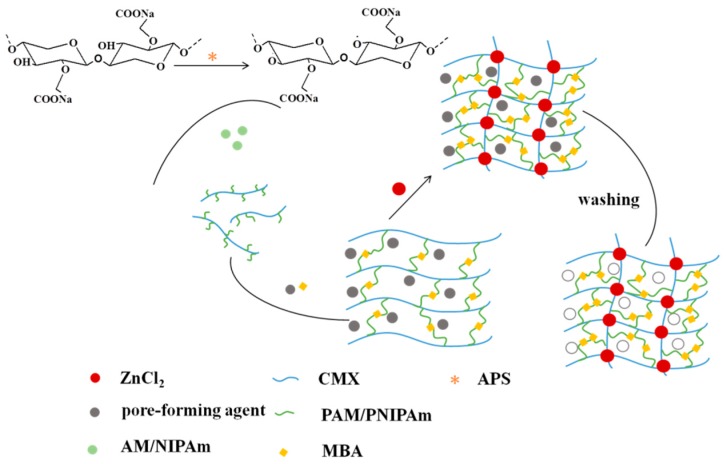
The preparation process of hydrogels.

The preparation process of the hydrogels is depicted in Scheme 1. The hydroxyl groups of CMX generated free radicals as the active site in the presence of APS, by which AM and NIPAm were grafted onto the CMX chain. AM and NIPAm could form polyacrylamide (PAM), polynitroisopropyl acrylamide (PNIPAm) and P(AM-co-NIPAm) under the function of APS. Then, the expected CMX-g-P(AM-co-NIPAm) hydrogels were formed under the function of MBA. Subsequently, the hydrogels were immersed in ZnCl_2_ solution to further enhance the strength of the hydrogels by coordination bonds formed between Zn^2+^ and anionic groups such as –COO^−^ [29]. During the process of hydrogel formation, the action of the pore-forming agents did not involve this chemical reaction. In view of the pore-forming agent type, different methods were used to remove pore-forming agents (Table 1). Thus, hydrogels with different pore sizes could be obtained.

### 3.2. FTIR Analysis of Hydrogels

**Figure 1:**
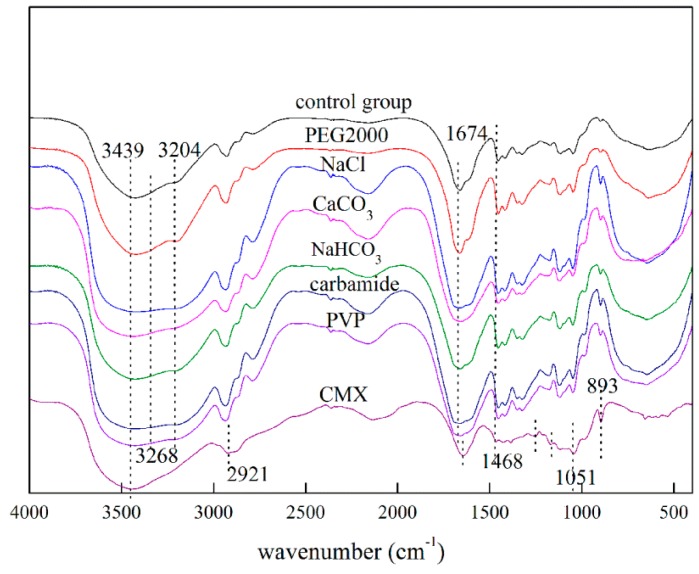
FTIR spectra of CMX and prepared hydrogels with or without six kinds of pore-forming agents.

The FTIR spectra of CMX and CMX-based hydrogels with six kinds of pore-forming agents are illustrated in Figure 1. For the spectrum of CMX, the broad band at 3439 cm^−1^ is assigned to the –OH groups on xylan. The bands appearing at 1646, 1254, and 1170 cm^−1^ are the typical absorption bands of xylan [30]. The band at 2921 cm^−1^ is assigned to the C–H stretching vibration of the alkane on xylan. The band at 1051 cm^−1^ originates from the C–O–C stretching of the pyranoid ring of xylan [22]. The sharp peak appearing at 893 cm^−1^ is owing to the β-glucosidic bonds between the xylose units. The absorption peak appearing at 1468 cm^−1^ is associated with the –COO^−^ symmetric stretching vibration of CMX [19]. For the spectrum of the hydrogels, the band at 3268 cm^−1^ originates from the N–H asymmetric stretching vibration peak of the carbonyl in the amide group in PNIPAm and PAM [31]. A peak at 3204 cm^−1^ is attributable to –NH_2_ stretching in PAM molecules [32]. The absorption peak at 1674 cm^−1^ is associated with the C=O stretching vibration peak of PAM and PNIPAm [33]. There is also an asymmetrical stretching band of carboxylate ions from 1468 cm^−1^ to 1457 cm^−1^ [34]. The FTIR spectra indicate that the monomers were grafted onto the structure of xylan. In addition, similar spectra of hydrogels with or without pore-forming agents were observed, indicating that organic pore-forming agents were completely removed during the elution stage and had no effects on the structure of the hydrogels.

### 3.3. Mechanical Properties Analysis of Hydrogels

**Figure 2:**
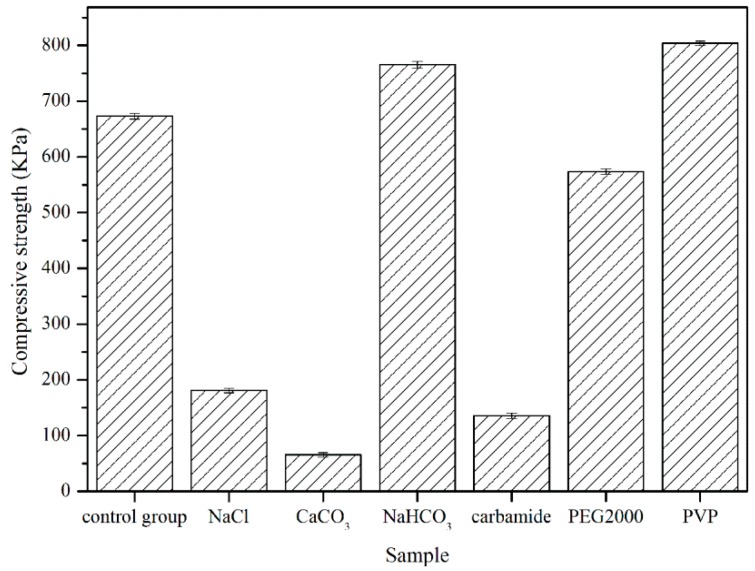
Compressive properties of hydrogels with or without six kinds of pore-forming agents.

Hydrogels should be endowed with suitable strength to broaden their applications. The compressive property was used to evaluate the mechanical properties of the hydrogels. Without the immersion of the hydrogels in ZnCl_2_ solution, breakable hydrogels were obtained, and the testing of the mechanical properties of the hydrogels could not be conducted, limiting further applications. To substantially improve the strength of the hydrogels, ZnCl_2_ was employed to facilitate the formation of strong cross-linked bonds (metal coordination bonds) between Zn^2+^ and –COO^−^ groups in the hydrogels [29]. After immersion in ZnCl_2_, the hydrogels had great elasticity and were not broken after pressing with fingers. The compressive strengths of the hydrogels with or without the six kinds of pore-forming agents are illustrated in Figure 2.

The compressive strength of the CMXH hydrogels without pore-forming agents (control group) was about 673 KPa. Different compressive strengths were obtained for hydrogels with PVP, PEG2000, carbamide, NaCl, CaCO_3_, and NaHCO_3_. The highest achieved compressive strength of hydrogels was 803 KPa with PVP, followed by hydrogels with NaHCO_3_, PEG2000, NaCl, carbamide, and CaCO_3_ (765 KPa, 573 KPa, 181 KPa, 135 KPa, and 65 KPa, respectively). This difference was ascribed to the use of pore-forming agents during the formation of the hydrogels. For organic pore-forming agents, the compressive strength of hydrogels with PVP was highest due to the molecular weight and the chain length. The entanglement of PVP with CMX or other polymers increased the crosslinking density. However, the high crosslinking density limited the increase in the pore size. The compressive strength of the hydrogel with PEG2000 was lower than that of CMXH hydrogels. This might be related to pore shrinkage because ethanol was used during the process of removal of the pore-forming agents, resulting in low mechanical properties compared with the CMXH hydrogels (control group). The compressive strength of the hydrogel with carbamide was about 140 KPa. One possible explanation for this is that hydrogen bonds were formed between carbamide and monomers, which reduced the cross-linking density of the hydrogels.

The lowest compressive strength among the hydrogels was obtained by using CaCO_3_ as the pore-forming agent. A possible reason for this is that the CaCO_3_ was not dissolved in water, so CaCO_3_ precipitate inhibited the effect of ZnCl_2_ in cross-linking with the hydrogels. When the pore-forming agent was NaHCO_3_, the compressive strength was relatively higher compared to the control group. NaHCO_3_ is water soluble and was well distributed inside the hydrogels. During the removal of NaHCO_3_ using HCl solution, no negative effect on the formation of crosslinking bands within the network of hydrogels was observed, unlike the situation for CaCO_3_. The crimp phenomenon of CMX occurred in the salt solution, which weakened the effect of Zn^2+^ [35], so a lower compressive strength for the hydrogel with NaCl was observed. Therefore, the molecular weight and chain length of the pore-forming agents, the pore size of the hydrogels, and the methods of removal of pore-forming agents had an important influence on the compressive strength of the hydrogels.

### 3.4. TGA Analysis of Hydrogels

**Figure 3:**
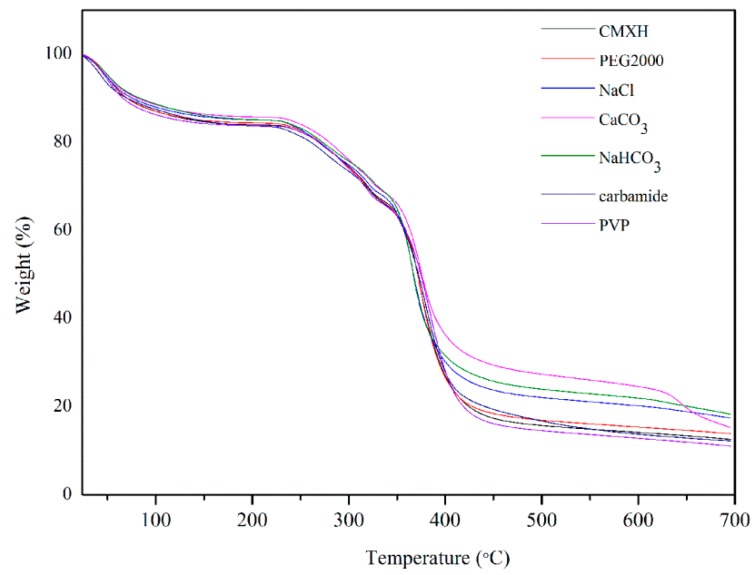
Thermogravimetric curves of the seven kinds of hydrogels.

The thermogravimetric curves reflected the thermal stability of the materials as well as the structural changes to some extent. Figure 3 shows the thermogravimetric curves of different hydrogels, and Figures S1–S7 show the thermogravimetric curves of the seven kinds of hydrogels. TGA analysis was used to evaluate the thermal stability of the hydrogels. The whole degradation process could be divided into three stages as follows. The weight loss process which occurred at 20–200 °C was ascribed to the loss of free water and the water of crystallization in the hydrogels. Weight loss between 200 °C and 400 °C was due to the breaking of intermolecular hydrogen bonds and the degradation of mainly CMX molecular chains and PAM and PNIPAm molecular chains [31]. After 400 °C, the weight of the samples was mainly related to the carbonization process of the polymer matrix. The residual carbon rate of the hydrogels was in the range of 10–18%. Obviously, there were similar thermogravimetric curves for all hydrogels before 400 °C, indicating that the pore-forming agents had little effect on the stability of the hydrogels. Above 400 °C, the difference was due to inorganic pore-forming agents. During the method of removal of pore-forming agents, it was difficult to remove all inorganic agents because they can be deposited on the surface of the hydrogels.

### 3.5. SEM Analysis of Hydrogels

**Figure 4:**
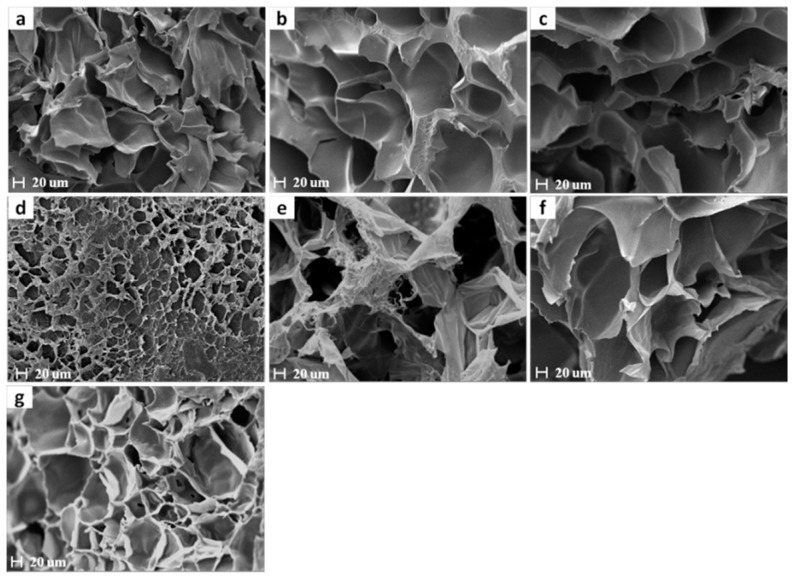
SEM images of hydrogels with (**a**) NaCl, (**b**) CaCO_3_, (**c**) NaHCO_3_, (**d**) PEG2000, (**e**) carbamide, (**f**) PVP, or (**g**) no pore-forming agents.

Figure 4 presents SEM images of the surface morphologies of the obtained hydrogels. Obvious network structures were observed for the hydrogels. The pore-forming agents had a remarkable influence on the pore sizes of the hydrogels. Hydrogels without a pore-forming agent (Figure 4a) had relatively small and nonuniform pores. Hydrogels with PEG2000 exhibited a smaller pore size in the range of 15–20 µm, which may be attributed to the PEG increasing the crosslink density [36]. Ethanol was used to dissolve PEG2000. The shrinkage of hydrogels in ethanol reduced the pore size and damaged the pore structure, which resulted in the low mechanical properties and swelling behavior compared with the CMXH hydrogels (control group). The pore size of the hydrogel with carbamide was in the range of 90–110 µm. One possible reason for this is that the carbamide decreased the cross-linking density by forming hydrogen bonds with reactive substances, which led to the relatively large pore size of the hydrogels after the removal of carbamide. The pore size of the hydrogels with PVP was about 60–90 µm. The high molecular mass of PVP increased the molecular entanglement. When the PVP was removed by water, pores were formed. For hydrogels with NaCl, the pore size was about 90–100 µm. One possible reason for this is that carboxymethyl xylan curled up in the salt solution, which decreased the crosslinking density. The pore size of the hydrogels with CaCO_3_ was similar to that of the hydrogels with NaHCO_3_ and was in the range of 100–130 µm. When hydrogels were soaked in the HCl solution, the CO_2_ produced by CaCO_3_ and NaHCO_3_ increased the pore size [37].

### 3.6. Zn^2+^ Analysis of Hydrogels

**Figure 5:**
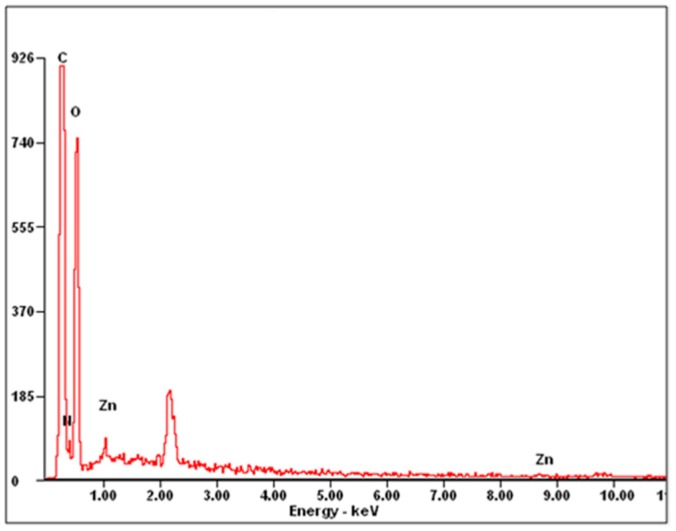
EDS spectrum of hydrogels without pore-forming agents.

Zinc is a highly essential element for humans and has a good biocompatibility, so it can be applied in biomedical applications [38]. ZnCl_2_ was used to increase the mechanical strength of hydrogels by interacting with carboxyl groups on CMX. An electron microprobe was used to confirm the presence of Zn^2+^ in hydrogels. Figure 5 showed the EDS of hydrogels without pore-forming agents. The EDS spectrum showed a Zn peak, indicating the existence of Zn in hydrogels. Table 2 shows the zinc content of the different hydrogels. The range of zinc contents in the hydrogels was about 0.09–0.3%, and the difference was cause by the pore-forming agents. The different contents were caused by pore-forming agents, suggesting that pore-forming agents had an impact on the metal coordination bonds between Zn^2+^ and –COO^−^ groups in the hydrogels.

### 3.7. Swelling Behavior Studies of Hydrogels

**Figure 6:**
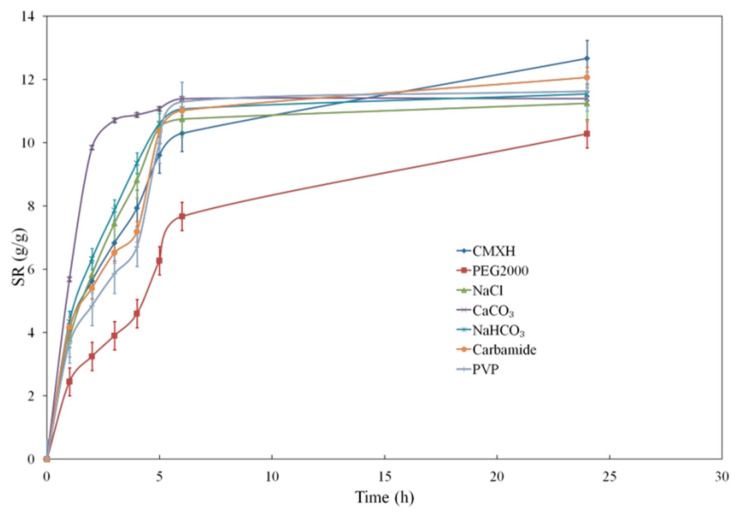
The swelling rates of hydrogels with or without six kinds of pore-forming agents in PBS.

The swelling capacity of hydrogels is important to their application. The swelling rate of hydrogels is related to their pore size [39]. Large pores in hydrogels are conducive to the diffusion of water molecules into the hydrogels [40]. Thus, hydrogels with larger pores had the faster swelling rate. The swelling ratio of the hydrogels in PBS solution as a function of time is shown in Figure 6. Hydrogels without any pore-forming agents did not reach swelling equilibrium after 24 h. In contrast, hydrogels with CaCO_3_ and NaHCO_3_ reached swelling equilibrium after 6 h, while hydrogels with PVP and NaCl reached swelling equilibrium within 24 h. Hydrogels with carbamide did not reach the swelling equilibrium within 24 h but had a higher SR than the hydrogels without any pore-forming agents. Hydrogels with PEG2000 could not reach the swelling equilibrium after 24 h and had a lower SR than the hydrogels without any pore-forming agents. According to the swelling rate of hydrogels in the PBS solution, hydrogels with CaCO_3_ and NaHCO_3_ had the biggest pore size. Hydrogels with PVP, carbamide, and NaCl had bigger pore size and hydrogels with PEG2000 had smaller pore size than hydrogels made without any pore-forming agents. These results correspond to the SEM results.

### 3.8. Drug Release of the Hydrogel Made with NaHCO_3_ Pore-Forming Agent

**Figure 7:**
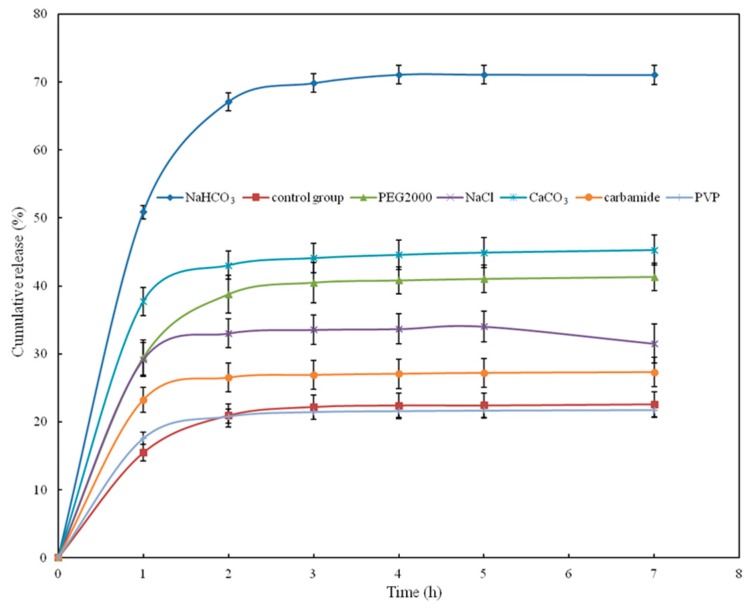
In vitro cumulative drug release from hydrogels in PBS solution (pH 7.4) at 37 °C.

5-Fu, which has been investigated as an antitumor agent used for treating solid tumors, was chosen as the drug model in this work. In this work, hydrogels with or without six kinds of pore-forming were applied to study the 5-Fu drug release performance, which is shown in Figure 7. Obviously, for all the hydrogels, the cumulative drug release rate increased rapidly over the first hour, and the cumulative drug release then increased slowly. After 4 h, the cumulative release remained constant. The drug release of hydrogels with NaHCO_3_ could reach up to 71.05% after 4 h, while the drug release of hydrogels with CaCO_3_ was 42.28% and the drug release of hydrogels without pore-forming agents only reached 22.57%. The drug release of the other hydrogels was about 21–41%. These results imply that hydrogels made with NaHCO_3_ have the high release rate for 5-Fu. Thus, the drug release properties of a hydrogel prepared using pore-forming agents could be significantly improved compared to a hydrogel without pore-forming agents. Suitable pore-forming agents gave rise to the enhancement of the drug release properties of the hydrogels due to the introduction of desirable pores within the network of the hydrogels. As shown in Figure 7, drug release balance was achieved after four hours in simulated intestinal fluid (pH = 7.4), and the maximum cumulative release rate achieved was 71.05%, implying that this hydrogel would have potential applications as a drug carrier.

## 4. Conclusions

In this work, CMX-based hydrogels with macroporous structure were prepared, and different inorganic or organic pore-forming agents were applied to study their influence on the pore size of the hydrogels. For organic pore-forming agents (PEG2000, carbmmide, PVP), the molecular weight and the chain length had an important influence on the pore size, the compressive strength, and the swelling ratio. For inorganic pore-forming agents (NaCl, CaCO_3_, NaHCO_3_), hydrogels with NaHCO_3_ displayed great performance in terms of the pore size of the hydrogels, mechanical properties, and drug release. Moreover, the pore-forming agents had little influence on the thermal stability of the hydrogels. The cumulative drug release for 5-Fu-loaded hydrogels made with NaHCO_3_ could reach up to 71.05%, which suggests that these hydrogels with macroporous structure have potential applications in the field of drug release. In the follow-up experiment, we will focus on the influence of different amounts of NaHCO_3_ on the properties of xylan-based hydrogels, as well as their applications to drug delivery.

## Figures and Tables

**Table 1:** Preparation conditions of hydrogels with different pore-forming agents.

Sample	Pore-Forming Agent	Washing Method
gel-1	—	Soaked with ultra-pure water for one week
gel-2	PEG2000	Soaked with ethanol for three days then ultra-pure water for four days
gel-3	NaCl	Soaked with ultra-pure water for one week
gel-4	CaCO_3_	Soaked with HCl solution for three days then ultra-pure water for four days
gel-5	NaHCO_3_	Soaked with HCl solution for three days then ultra-pure water for four days
gel-6	carbamide	Soaked with ultra-pure water for one week
gel-7	PVP	Soaked with ultra-pure water for one week

**Table 2:** Zinc content in the different hydrogels.

Sample	gel-1	gel-2	gel-3	gel-4	gel-5	gel-6	gel-7
Hydrogel Weight (mg)	33.80	50.80	71.50	43.40	63.90	43.60	51.50
Zinc Concentration (mg/L)	7.18	15.02	14.44	4.17	11.32	5.78	14.28
Weight Percent of Zinc (%)	0.21	0.29	0.20	0.09	0.18	0.13	0.27

## Supplementary Materials

The following are available online at http://www.mdpi.com/1999-4923/10/4/261/s1, Figures S1–S7: Thermogravimetric curves of hydrogels with PEG2000, NaCl, CaCO_3_, NaHCO_3_, carbamide, and PVP, and of hydrogels without pore-forming agents.
