# Supplementary material for: Xylan-Based Hydrogels as a Potential Carrier for Drug Delivery: Effect of Pore-Forming Agents
Source: Pharmaceutics. 2018 Dec 5;10(4):261. doi: 10.3390/pharmaceutics10040261 (PMC6321516; doi:10.3390/pharmaceutics10040261)
Supplement: Supplementary file 1 [file pharmaceutics-10-00261-s001.pdf]

pharmaceutics

Supplementary Materials: Xylan-Based Hydrogels as a Potential Carrier for Drug Delivery: Effect of Pore-Forming Agents

Minmin Chang, Xinxin Liu, Ling Meng, Xiaohui Wang and Junli Ren

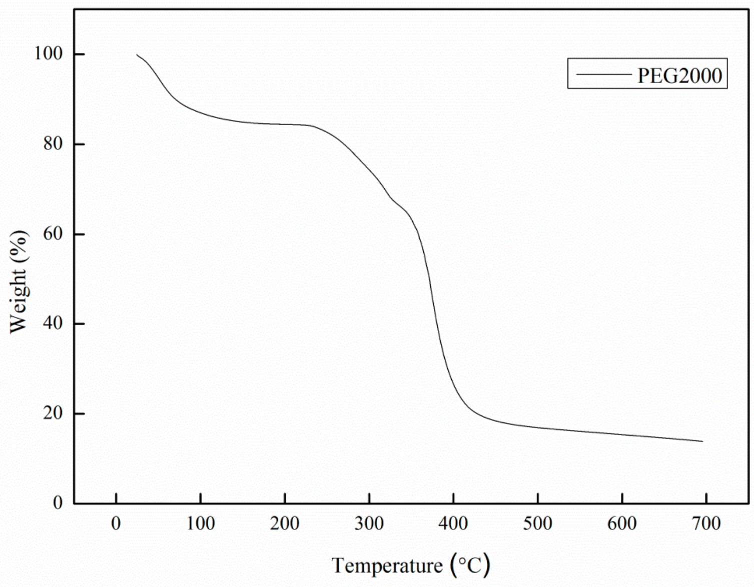

Figure S1. Thermogravimetric curve of hydrogels with PEG2000.

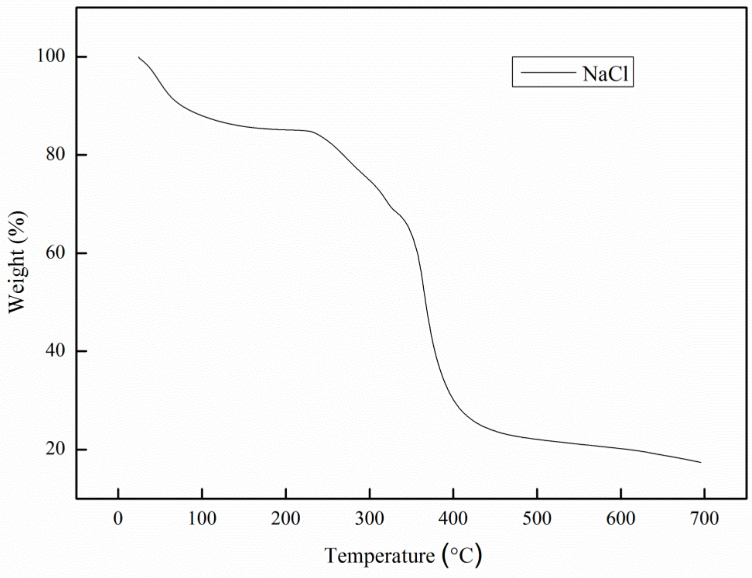

Figure S2. Thermogravimetric curve of hydrogels with NaCl.

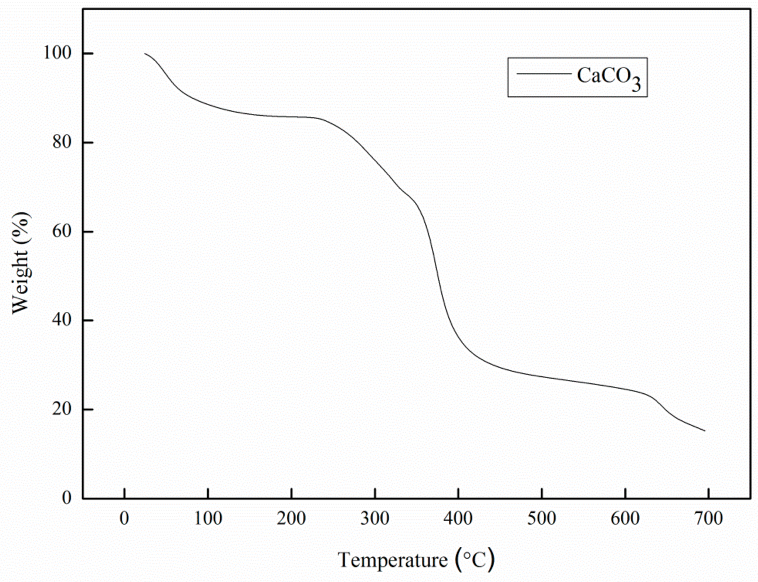

Figure S3. Thermogravimetric curve of hydrogels with $CaCO_3$.

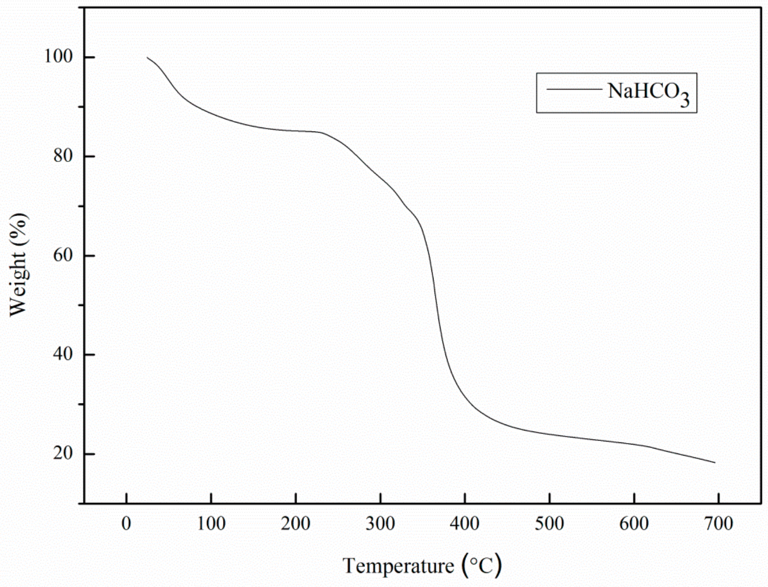

Figure S4. Thermogravimetric curve of hydrogels with $NaHCO_3$.

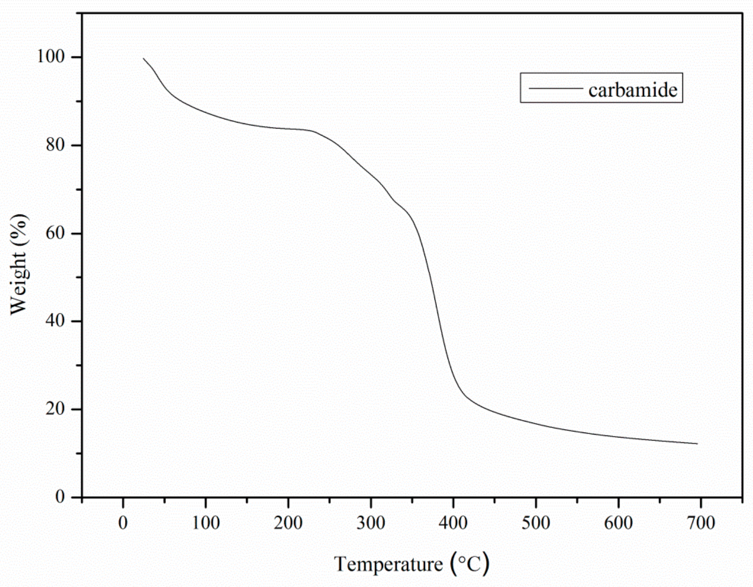

Figure S5. Thermogravimetric curve of hydrogels with carbamide.

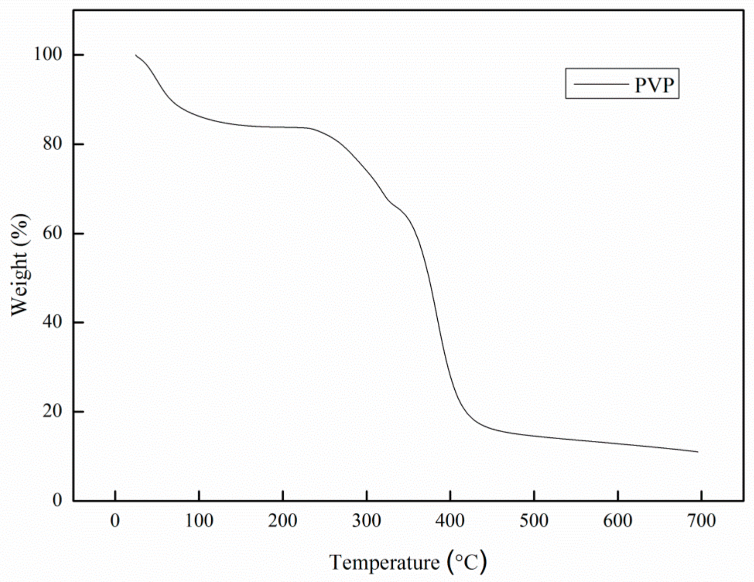

Figure S6. Thermogravimetric curve of hydrogels with PVP.

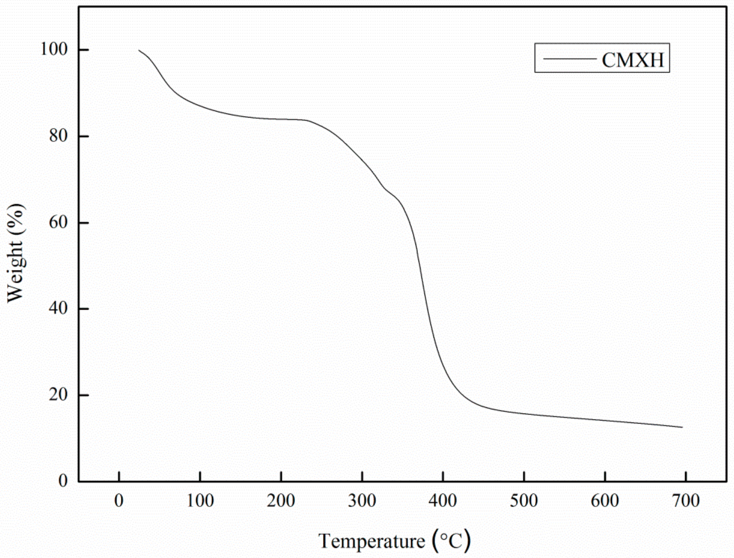

Figure S7. Thermogravimetric curve of hydrogels without pore-forming agents.
